# Association between perceived neighborhood environment and depression among residents living in mega-communities in Guiyang, China: a cross-sectional study

**DOI:** 10.1186/s12889-024-17844-z

**Published:** 2024-02-01

**Authors:** Yong Lu, Zenglin Li, Kai Qin, Jiao Chen, Nana Zeng, Bo Yan, Di Liu

**Affiliations:** 1 School of Public Health, the Key Laboratory of Environmental Pollution Monitoring and Disease Control, Ministry of Education, Guizhou Medical University, Guiyang, China; 2 Guangzhou Medical University Library, Guangzhou, China; 3 School of Public Health, Guangzhou Medical University, Guangzhou, China

**Keywords:** Perceived neighborhood environment, Depression, Mega-community, Mainland China, Structural equation model

## Abstract

**Background:**

Little was known about the relationship between perceived neighborhood environment and depression among residents living in mega-communities. Furthermore, the mediating effects of physical activity (PA) and anxiety in this relationship have not been investigated. Thus, this study aimed to comprehensively examine the association between perceived neighborhood environment and depression among residents living in mega-communities, and test whether PA and anxiety mediated the association.

**Methods:**

A cross-sectional study on perceived neighborhood environment and depression was conducted among individuals who lived in mega-communities (*n* = 665) in Guiyang, China from July to August 2022. Perceived neighborhood environment was assessed from the following six aspects: traffic, building quality, accessibility, neighborhood, indoor, and pollution. Depression was measured by the Patients Health Questionnaire-9. Structural equation model was used to evaluate the association between perceived neighborhood environment and depression, and test the mediating effect of PA and anxiety in this association.

**Results:**

We found that neighborhood (*β* = -0.144, *p* = 0.002) and PA (*β* = -0.074, *p* < 0.001) were both negatively associated with depression, while anxiety was positively associated with depression (*β* = 0.447, *p* < 0.001). Married residents were less likely to experience depression than residents of other marital status. PA played a mediator role in the relationship between accessibility and depression (*β* = 0.014, *p* = 0.033). PA mediated the relationship between neighborhood and depression (*β* = -0.032, *p* = 0.015). The mediating effect of anxiety in the relationship between perceived neighborhood environment and depression was not significant.

**Conclusions:**

This study demonstrated that neighborhood, which was assessed by satisfaction with safety, hygiene, parking, greening, lighting, and building shape, was negatively associated with depression, and PA mediated the relationship.

**Supplementary Information:**

The online version contains supplementary material available at 10.1186/s12889-024-17844-z.

## Introduction

Depression is a common mental disorder that involves persistent sadness, loss of pleasure in activities, decreased energy, feelings of guilt or low self-worth, and difficulty concentrating [1, 2]. Data from the World Health Organization (WHO) indicated that approximately 280 million people (including the children and elderly) suffered from depression across the world in 2019, with a prevalence of 3.8% [3, 4]. The China Mental Health Survey carried out between 2012 and 2015 showed that the lifetime prevalence of depressive disorders among Chinese adults was 6.8% [5]. Depression is an important contributor to the global burden of disease [6]. Several previous studies have shown that depression could increase the risk of a variety of chronic diseases, including cardiovascular diseases, hypertension, diabetes, and cancers [7–11].

With rapid economic development and policy liberalization, mainland China has been undergoing an unprecedented urbanization process since the 1990s [12]. From 1990 to 2022, the urbanization rate in China increased from 26.41% to 65.22% [13]. However, the rapid urbanization has brought a series of problems, such as overcrowding, urban housing shortages, and land resource scarcity [12, 14, 15]. To solve the problems mentioned above, a large number of mega-communities (e.g., Tiantongyuan in Beijing, Phoenix City in Guangzhou, and Century City in Guiyang) have emerged in many cities in mainland China [16, 17]. In this study, urban communities with a land area of more than 5 square kilometers and a permanent population of more than 100,000 are considered mega-communities [18]. In order to accommodate a large number of urban immigrants on limited land, most mega-communities are dominated by high-rise, high-density residential buildings [19]. The built form of mega-community has resulted in a variety of problems in neighborhood environment such as poor air ventilation, poor sunlight penetration, unsatisfactory air quality [20], inadequate outdoor activity space and green space, parking issues, etc. [21]. Besides, most of mega-communities in mainland China were developed and constructed in the last two decades. The medical and educational resources in these communities have yet to meet the needs of the residents living in these communities [16]. Additionally, most residents of mega-communities in mainland China are mainly low- and middle-income individuals (including migrants), which leads to many problems in the management and security of mega-communities (e.g., high crime rate) [16]. These unpleasant neighborhood conditions have been considered as environmental risk factors which might threaten the mental health of community residents [20, 21].

There is a growing body of research that indicates the relationships between the characteristics of perceived neighborhood environment (e.g., quality of built environment, noise, air pollution, green space, etc.) and depression. A cross-sectional study among adult residents in the United States (US) has shown that low quality of the built environment was found to be associated with the increased likelihood of depression [22]. Residential noise was found to be associated with an increased risk of symptoms of depression in a cohort study among randomly selected Germans aged 45 to 75 [23]. In the Netherlands, a pooled analysis of eight cohort studies demonstrated that higher air pollution, less green space, and less social security were related to higher rates of depression [24]. A cohort study among adult Turin residents suggested that good accessibility to public transport was related to the decreased risk of depression [25]. A cross-sectional study among the elderly in Shanghai, China indicated that the perceived neighborhood environment, comprehensively assessed by community safety, health care, and public transportation, was negatively associated with depression [26]. Another cross-sectional study conducted among Hong Kong residents aged 16 years or older revealed that crowded indoor living space and high residential building density might play a role in the development of depression [27].

Several studies exploring the associations between neighborhood environmental factors and depression have indicated that physical activity (PA) and anxiety played a mediating role in these associations. A cross-sectional study among African Americans revealed that PA was a mediator in the positive relationship between perceived neighborhood environment and depressive symptoms [28]. A study conducted by Pun VC et al. showed that the association between urbanization and depressive symptoms among older residents in the US was mediated by PA [29]. A study aimed at older residents in Hong Kong also demonstrated PA was a mediating variable in the relationship between neighborhood environmental factors and depressive symptoms [30]. In Spain, de la Torre-Cruz et al. found that anxiety was a mediating variable in the relationship between self-esteem and depression [31]. Several studies in China also reported that anxiety mediated the relationship between influence factors (e.g., social support and cyberbullying perpetration) and depression [32, 33].

Earlier studies have reported that perceived neighborhood environment played a role in the development of depression among community residents [23, 34–36]. However, several research gaps still exist in this field. Firstly, previous studies focusing on mega-communities in mainland China have indicated that there are many problems in the neighborhood environment of mega-communities, e.g., lack of public space, lack of security and comfort [37], inconvenient transportation, insufficient public services, and inadequate medical and educational resources [16]. However, little was known about the association between perceived neighborhood environment and depression among residents living in mega-communities in mainland China. Secondly, previous studies have always focused on the relationships between one specific or limited number of environmental factors (e.g., green space, accessibility to public transport, and living density) and depression [24, 25, 27], and few studies have comprehensively assessed the relationship between perceived neighborhood environment and depression. Finally, to the best of our knowledge, whether PA and anxiety mediated the relationship between perceived neighborhood environment and depression among residents living in mega-communities in mainland China is still unknown.

Our primary aim was to test the hypothesis that perceived neighborhood environment was negatively associated with depression among residents living in mega-communities in mainland China. The secondary aim was to test the hypothesis that PA and anxiety mediated the relationship between perceived neighborhood environment and depression among residents living in mega-communities in mainland China, while controlling for socio-demographic characteristics.

## Materials and methods

### Setting and participants

This was a cross-sectional study conducted in mega-communities in Guiyang, Southwest China from July to August 2022. The standard of a mega-community is a community with a land size of more than 5 square kilometers and a permanent population of more than 100,000 [18]. There are three mega-communities which meet the above criteria in Guiyang: Flower Orchard, Future Ark, and Century City [16].

Multi-stage cluster random sampling strategy was used to obtain the participants. A mega-community would be divided into several *zutuans*. Each *zutuan* contains several residential buildings. *Zutuan* is an intermediate unit between the community and the residential building [38]. In the first stage, 12 *zutuans* were randomly selected from the 104 *zutuans* in the three mega-communities. There were 150 residential buildings in the selected *zutuans*. In the second stage, 38 residential buildings were randomly selected. In the third stage, we randomly invited a household from each floor of the selected residential buildings to participate. In the last stage, we randomly selected a participant from eligible family members of each selected household (i.e., only one eligible family member from each selected household would participate in this survey). Simple random sampling without replacement was performed using pseudorandom number generator in combination with a list the *zutuans*, residential buildings, and eligible family members. The three mega-communities yielded a potential pool of 1078 households, among which 700 participants completed our questionnaire. We required that participants fulfilled the following inclusion criteria: (1) to be aged 14 or older, and (2) living in a mega-community for at least six months. The exclusion criteria were as follows: (1) not providing informed consent; and (2) not understanding the questionnaire.

The ethical approval of this study was obtained from the Ethics Review Committee of Guizhou Medical University (No.2021–082). All participants in this study provided written informed consent before participating in this study.

### Measuring instruments

The paper questionnaire used in this study consisted of the following five sections: socio-demographic characteristics, perceived neighborhood environment, anxiety, PA, and depression. The questionnaire is attached as a supplement.

Socio-demographic characteristics obtained in this study included the following variables: gender, age, education degree (college or above, lower than college), marital status (married; single, divorced, separated, or widowed), employment status (employed, unemployed), per capita monthly income (RMB) (≤2000, 2001–5000, 5001–8000, 8001–15000, >15,000) [39], local household registration (yes, no), homeowner (yes, no), years living in this community (year), number of family member, and overall house area (m^2^). Household registration is an identification for many Chinese citizens. Only citizens with local household registration can enjoy welfare provided by local government [40]. Average living space per person (m^2^) is calculated by dividing the overall house area by the number of family members.

Perceived neighborhood environment referred to perceived satisfaction with the neighborhood environment which was comprehensively assessed by items relating to the following six themes: traffic, building quality, accessibility, neighborhood, indoor, and pollution [41]. All the items were presented as follows: “How satisfied are you with … in your community”. Perceived satisfaction is the subjective evaluation of the performance of a given environmental factor, indicating to which extent the given environmental factor meets the expectations and needs of the residents [42, 43]. The traffic subscale was used to evaluate satisfaction with traffic conditions within a community, including six items assessing the distance to the main road, road condition, sidewalk, road connectivity, traffic safety, and traffic congestion. The building quality subscale was used to assess satisfaction with quality of residential buildings in a community, including five items assessing the quality of elevators, doors and windows, building envelope (e.g., external walls, roofs, and floors), building structure, and building facilities (e.g., pipes, pumps, and water tanks). The accessibility subscale was used to measure satisfaction with ease of reaching needed or desired activities within a community, using five items to inquire about the accessibility to parks and green land, medical institutions, entertainment venues, transportation stations, and shopping malls. The neighborhood subscale was used to evaluate satisfaction with public facilities and public environment within a community, where six items were asked on neighborhood safety, hygiene, parking, greening, lighting, and building shape. The indoor subscale was used to assess satisfaction with indoor environment (i.e., conditions inside the residential buildings), using four items evaluating the noise, ventilation, temperature, and lighting. The pollution subscale was used to measure satisfaction with environmental pollution within a community, including two items on air pollution and other pollution (e.g., water). All the above scales were measured on a five-point Likert scale ranging from 1 (strongly dissatisfied) to 5 (strongly satisfied). The higher scores indicated a more satisfied perception of neighborhood environment. The previous study which developed the perceived neighborhood environment scale has indicated that the reliability and validity of this scale were acceptable [41].

We used the Patients Health Questionnaire-9 (PHQ-9) to measure the depression of participants [44, 45]. The PHQ-9 has been widely used in depression measurement, which was composed of nine items to measure negative feelings (such as “Feeling down, depressed or hopeless”) within the last two weeks.

The Generalized Anxiety Disorder-7 (GAD-7), a self-rating scale, was chosen to evaluate anxiety [46]. The GAD-7 consisted of seven items to assess anxiety disorder and anxiety associated symptoms over the last two weeks (such as “Not being able to stop or control worrying”). The PHQ-9 and GAD-7 were measured on a four-point Likert scale ranging from 0 (not at all) to 3 (nearly every day). The total score of PHQ-9 ranged between 0 and 27, and a higher score indicated more serious depression. Participants with a total PHQ-9 score of 0 to 4 were considered as not reporting depressive symptoms, and participants with a total PHQ-9 score of 5 or above were considered as reporting depressive symptoms [47]. The total score of GAD-7 was from 0 to 21, and a high score revealed more severe anxiety [48]. Previous studies have revealed the high reliability and validity of PHQ-9 and GAD-7 [49–51].

PA was assessed by the short form of the International Physical Activity Questionnaire (IPAQ-S) which has been proved to have satisfied reliability and validity [52, 53]. IPAQ-S was comprised of seven items to collect information on the duration, frequency, and intensity of PA (i.e., walking, moderate-intensity PA[MPA], and vigorous-intensity PA[VPA]) over the last week. One metabolic equivalent (MET) was defined as the amount of oxygen one person consumed while sitting at rest [54]. In order to calculate a continuous MET for each participant, a MET value was assigned to each type of PA. According to the guideline for IPAQ-S, MET values for walking, MPA, and VPA were 3.3, 4.0, and 8.0, respectively [55]. MET for each type of PA per week was calculated by the MET value times the minutes spent on that PA per day times the number of days that the PA was carried out per week (e.g., VPA MET = 8.0 × daily time spent × frequency per week) [52]. The total MET of PA was calculated as the sum of the METs of the three specific PAs. According to the scoring protocol of IPAQ-S, we divided the participants into three PA intensities: low, moderate, and high [56].

### Statistical analyses

We divided all the participants into the depression group and the non-depression group based on the PHQ-9 scores. Participants with a PHQ-9 score higher than 4 were assigned to the depression group, while the rest were assigned to the non-depression group. We conducted descriptive analyses on the socio-demographic characteristics, perceived neighborhood environment, and anxiety. For categorical variables, frequencies and percentages were used. For normally distributed continuous variables, *mean* and *standard deviation* (*SD*) were calculated, while for non-normally distributed continuous variables, *median* and *interquartile ranges* (*IQR*) were calculated. *Chi-square* test, *Student’s t* test and *Wilcoxon* test were used for comparing categorical variables, normally distributed continuous variables and non-normally distributed continuous variables.

**Fig. 1:**
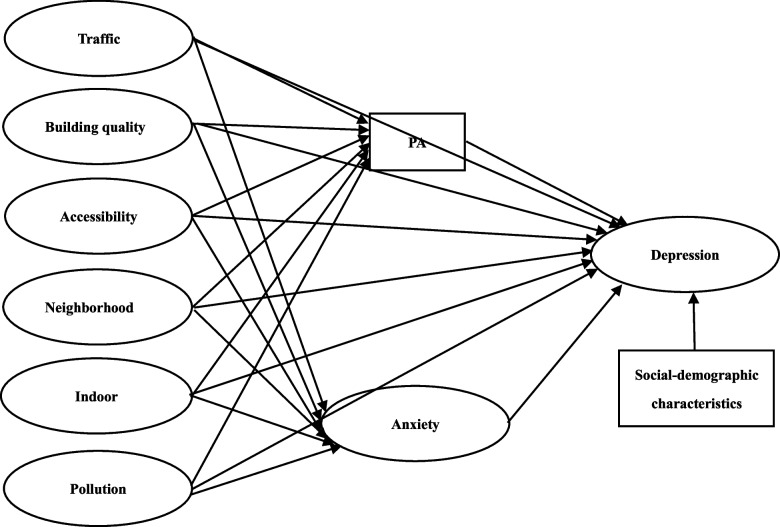
The theoretical model

Then, the structural equation model (SEM) was used to examine the relationship between perceived neighborhood environment and depression, and test whether PA and anxiety mediated this relationship among residents living in mega-communities, while controlling for socio-demographic characteristics. SEM is a combination of factor analysis and multiple regression analysis, which is mainly used to simultaneously estimate the structural associations between manifest variables and latent constructs. It is one of the widely used methods for examining mediating effects. Meanwhile, SEM provides statistical indicators to assess the goodness of fit of the model [57]. The above-mentioned advantages of SEM were consistent with the objectives of our study. The theoretical model of this study is shown in Fig. 1. The relationships among the above variables were estimated using the Generalized Least Squares estimation [58]. Mediating effects were estimated as the product of the direct effect coefficients [59] and tested using the bootstrapping method [60]. Indicators used to evaluate the goodness-of-fit of the model were as follows: discrepancy divided by degree of freedom (CMIN/DF), root mean square error of approximation (RMSEA), incremental fit index (IFI), Tucker-Lewis incremental fit (TLI), comparative fit index (CFI), normed fit index (NFI), adjusted goodness of fit index (AGFI), goodness of fit index (GFI). When CMIN/DF was less than 5, RMSEA was less than 0.08, and IFI, TLI, CFI, NFI, AGFI, and GFI were all greater than 0.90, the model was considered to be adequate [61]. The descriptive analyses and comparisons between the two groups were performed using SPSS 21.0, and the SEM was fitted using SPSS AMOS 26. Two-tailed *P* value less than or equal to 0.05 was considered statistically significant.

## Results

### Description of socio-demographic characteristics

A total number of 1078 paper questionnaires were distributed, among which 700 were returned, resulting in a response rate of 64.9%. After carefully reviewing, 35 questionnaires were found to be incomplete. Finally, 665 participants with valid responses were included in this study. We compared the sociodemographic information between participants with valid responses and participants with invalid responses and no statistically significant were observed.

The socio-demographic characteristics of participants are listed in Table 1. The participants were composed of 273 (41.1%) males and 392(58.9%) females, with an average age of 41.4 (± 17.9) years old. Among all participants, 444(66.8%) were married and 405 (60.9%) were unemployed. About half (48.3%) of the participants had a college degree or above. There were 416 (62.6%) participants that owned local household registration and 585 (88.0%) participants were homeowners. The majority (70.6%) of participants had a per capita monthly income of 2000–8000 RMB. High PA was reported by 282(42.4%) participants, while 194(29.2%) and 189 (28.4%) reported low and moderate PA. For all participants, the average body mass index (BMI) was 22.3 ± 3.1 kg/m^2^, and the average years living in mega-community was 5.0 ± 2.7 years. In addition, the median living area per person was 30.0 m^2^.

**Table 1:** Social-demographic characteristics and physical activity level of all participants by depression status

Characteristics	Full sample *N* = 665	Non-depression group *N* = 539	Depression group *N* = 126	*P*
	Mean ± SD	Mean ± SD	Mean ± SD	
Age (year)	41.4 ± 17.9	42.1 ± 18.3	38.3 ± 15.7	0.019
BMI (kg/m^2^)	22.3 ± 3.1	22.3 ± 3.0	22.3 ± 3.6	0.955
Years living in this community (year)	5.0 ± 2.7	5.0 ± 2.7	4.9 ± 2.9	0.802
	Median (Q_1_, Q_3_)	Median (Q_1_, Q_3_)	Median (Q_1_, Q_3_)	
Living area per person (m^2^)	30.0 (23.1, 39.0)	30.0 (22.5, 39.0)	30.0 (25.0, 39.4)	0.373
	*N* (%)	*N* (%)	*N* (%)	
Gender				0.884
Female	392 (58.9)	317 (80.9)	75 (19.1)	
Male	273 (41.1)	222 (81.3)	51 (18.7)	
Employment status				0.286
Employed	260 (39.1)	216 (83.1)	44 (16.9)	
Unemployed	405 (60.9)	323 (79.8)	82 (20.2)	
Marital status				0.055
Married	444 (66.8)	369 (83.1)	75 (16.9)	
Single, divorced, separated, or widowed	221 (33.2)	170 (76.9)	51 (23.1)	
Educational degree				0.408
College or above	321 (48.3)	265 (79.8)	65 (20.2)	
Lower than college	344 (51.7)	283 (82.3)	61 (17.7)	
Local household registration				0.971
Yes	416 (62.6)	377 (81.0)	79 (19.0)	
No	249 (37.4)	202 (81.1)	47 (18.9)	
Homeowner				0.141
Yes	585 (88.0)	479 (81.9)	106 (18.1)	
No	80 (12.0)	60 (75.0)	20 (25.0)	
Per capita monthly income (RMB)				0.295
≤2000	63 (9.5)	49 (77.8)	14 (22.2)	
2001–5000	235 (35.3)	198 (84.3)	37 (15.7)	
5001–8000	235 (35.3)	182 (77.4)	53 (22.6)	
8001–15000	100 (15.0)	82 (82.0)	18 (18.0)	
>15,000	32 (4.0)	28 (87.5)	4 (12.5)	
Physical activity				0.013
Low	194 (29.2)	144 (74.2)	50 (25.8)	
Moderate	189 (28.4)	156 (82.5)	33 (17.5)	
High	282 (42.4)	239 (84.8)	43 (15.2)	
Community				0.319
Flower Orchard	432 (65.0)	343 (79.4)	89 (20.6)	
Future Ark	133 (20.0)	111 (83.5)	22 (16.5)	
Century City	100 (15.0)	85 (85.0)	15 (15.0)	

The scores of perceived neighborhood environment and anxiety are presented in Table 2. The item mean scores of traffic, building quality, accessibility, neighborhood, indoor, and pollution ranged from 2.94 to 3.46, 3.08 to 3.49, 3.15 to 3.48, 3.04 to 3.37, 2.77 to 3.44, and 2.92 to 3.35, respectively. The score of anxiety items ranged from 0.18 to 0.42, and the mean total anxiety score was 2.21.

**Table 2:** Perceived neighborhood environment and anxiety of all participants by depression status

Items	Full sample *N* = 665	Non-depression group *N* = 539	Depression group *N* = 126	*P*
Perceived neighborhood environment				
Traffic				
Distance to the main road	3.46 ± 0.89	3.49 ± 0.87	3.33 ± 0.95	0.083
Road condition	3.27 ± 0.92	3.35 ± 0.90	2.94 ± 0.92	< 0.001
Sidewalk	3.00 ± 0.94	3.04 ± 0.93	2.85 ± 0.96	0.039
Road connectivity	3.36 ± 0.89	3.40 ± 0.90	3.19 ± 0.83	0.013
Traffic safety	3.39 ± 0.89	3.44 ± 0.89	3.19 ± 0.88	0.005
Traffic congestion	2.94 ± 0.93	2.98 ± 0.92	2.79 ± 0.96	0.039
Building quality				
Elevators	3.08 ± 0.98	3.13 ± 1.00	2.90 ± 0.89	0.017
Doors and windows	3.43 ± 0.86	3.47 ± 0.84	3.23 ± 0.91	0.004
Building envelope	3.20 ± 0.96	3.26 ± 0.96	2.95 ± 0.91	0.001
Building structure	3.38 ± 0.82	3.42 ± 0.81	3.22 ± 0.87	0.015
Building facilities	3.49 ± 0.80	3.53 ± 0.80	3.33 ± 0.79	0.012
Accessibility				
Parks and green land	3.15 ± 1.03	3.21 ± 1.02	2.88 ± 1.01	0.001
Medical institutions	3.38 ± 0.86	3.42 ± 0.87	3.20 ± 0.76	0.004
Entertainment venues	3.22 ± 0.92	3.27 ± 0.93	3.00 ± 0.83	0.002
Transportation stations	3.48 ± 0.87	3.50 ± 0.87	3.37 ± 0.88	0.128
Shopping malls	3.43 ± 0.88	3.46 ± 0.87	3.30 ± 0.91	0.070
Neighborhood				
Safety	3.31 ± 0.87	3.38 ± 0.86	3.01 ± 0.87	< 0.001
Hygiene	3.14 ± 0.98	3.20 ± 0.96	2.84 ± 1.03	< 0.001
Parking	3.04 ± 1.02	3.11 ± 1.03	2.75 ± 0.96	< 0.001
Greening	3.12 ± 0.99	3.17 ± 1.00	2.90 ± 0.92	0.003
Lighting	3.37 ± 0.87	3.44 ± 0.85	3.09 ± 0.91	< 0.001
Building shape	3.28 ± 0.81	3.35 ± 0.77	2.96 ± 0.87	< 0.001
Indoor				
Noise	2.77 ± 1.06	2.83 ± 1.05	2.48 ± 1.06	0.001
Ventilation	3.44 ± 0.86	3.50 ± 0.85	3.19 ± 0.88	< 0.001
Temperature	3.41 ± 0.84	3.47 ± 0.82	3.17 ± 0.87	< 0.001
Light	3.41 ± 0.93	3.50 ± 0.87	3.05 ± 1.07	< 0.001
Pollution				
Air pollution	3.35 ± 0.88	3.42 ± 0.85	3.06 ± 0.94	< 0.001
Other pollution (e.g., water)	2.92 ± 0.99	3.00 ± 0.98	2.62 ± 0.99	< 0.001
Anxiety				
Feeling nervous, anxious, or on edge	0.40 ± 0.71	0.26 ± 0.56	0.99 ± 0.93	< 0.001
Not being able to stop or control worrying	0.30 ± 0.64	0.16 ± 0.46	0.88 ± 0.94	< 0.001
Worrying too much about different things	0.42 ± 0.74	0.26 ± 0.57	1.10 ± 0.96	< 0.001
Trouble relaxing	0.34 ± 0.68	0.18 ± 0.49	0.98 ± 0.94	< 0.001
Being so restless that it is hard to sit still	0.19 ± 0.49	0.10 ± 0.36	0.57 ± 0.74	< 0.001
Becoming easily annoyed or irritable	0.39 ± 0.66	0.24 ± 0.51	1.04 ± 0.84	< 0.001
Feeling afraid, as if something awful might happen	0.18 ± 0.49	0.08 ± 0.30	0.63 ± 0.81	< 0.001
Total score of anxiety	2.21 ± 3.57	1.28 ± 2.41	6.19 ± 4.80	< 0.001

According to the total scores of PHQ-9, 126 (18.95%) participants reported symptoms of depression, while 539 (81.05%) participants did not report symptoms of depression. The difference in age between the depression and non-depression groups was statistically significant (*p* = 0.019). Participants who reported low PA showed the highest prevalence of depression (25.8%), followed by participants who reported moderate PA (17.5%) and high PA (15.2%), showing a significant difference (*p* = 0.013). No significant differences in other characteristics, including BMI, years living in this community, average living area per person, gender, employment status, marital status, educational degree, local household registration, homeowner, per capita monthly income, and community, were noted between the depression and non-depression groups (Table 1).

To explore potential factors associated with depression in perceived neighborhood environment, we performed comparison of perceived neighborhood environment items between the depression and non-depression groups. The results of comparison are listed in Table 2. There were significant differences between the two groups regarding “road condition” (*p* < 0.001), “sidewalk” (*p* = 0.039), “road connectivity” (*p* = 0.013), “traffic safety” (*p* = 0.005), “traffic congestion” (*p* = 0.039), “elevators” (*p* = 0.017), “doors and windows” (*p* = 0.004), “building envelope” (*p* = 0.001), “building structure” (*p* = 0.015), “building facilities” (*p* = 0.012), “parks and green land” (*p* = 0.001), “medical institutions” (*p* = 0.004), “entertainment venues” (*p* = 0.002), “safety” (*p* < 0.001), “hygiene” (*p* < 0.001), “parking” (*p* < 0.001), “greening” (*p* = 0.003), “lighting” (*p* < 0.001), “building shape” (*p* < 0.001), “noise” (*p* = 0.001), “ventilation” (*p* < 0.001), “temperature” (*p* < 0.001), “light” (*p* < 0.001), “air pollution” (*p* < 0.001), and “other pollution” (*p* < 0.001). Generally, the non-depression group presented higher perceived neighborhood environment scores than the depression group. In addition, all anxiety items and the average total score of anxiety differed significantly between the depression and non-depression groups (all *p* < 0.001). The depression group showed higher anxiety scores than the non-depression group. No significant differences in other perceived neighborhood environment items, including “distance to the main road”, “transportation stations”, and “shopping malls” were observed between the depression and non-depression groups.

### Test of study models

**Fig. 2:**
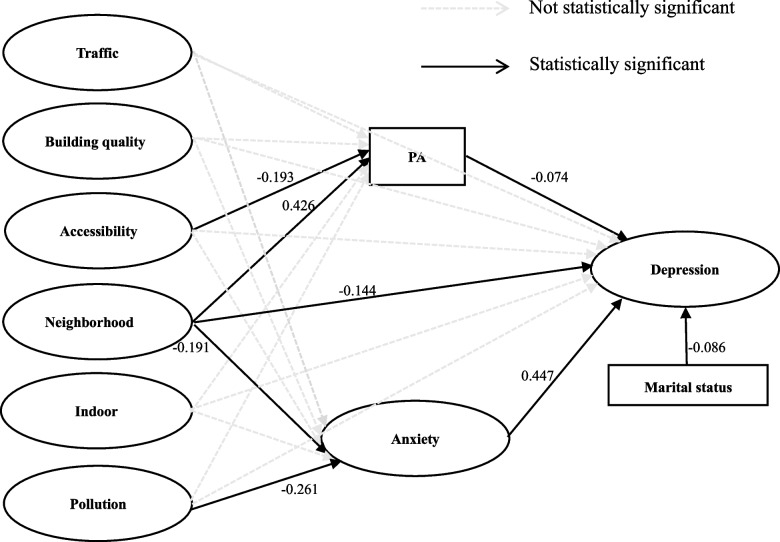
Final model of associations between perceived neighborhood environment and depression with mediators of physical activity and anxiety

The model fit indicators of the SEM shown in Fig. 2 were as follows: CMIN/DF = 1.162, GFI = 0.928, NFI = 0.916, CFI = 0.926, IFI = 0.932, TLI = 0.912, RMSEA = 0.016, and AGFI = 0.911, all of which indicated that the model fitted adequately.

Figure 2 showed the parameter estimates for the SEM. We found that accessibility was negatively associated with PA (*β* = -0.193, *p* = 0.026), while neighborhood was positively associated with PA (*β* = 0.426, *p* < 0.001). Pollution and neighborhood were negatively associated with anxiety (*β* = -0.261, *p* = 0.022; *β* = -0.191, *p* = 0.011). Anxiety was positively associated with depression (*β* = 0.447, *p* < 0.001), whereas PA (*β* = -0.074, *p* < 0.001) and neighborhood (*β* = -0.144, *p* = 0.002) were both negatively associated with depression. Additionally, marital status was negatively associated with depression (*β* = -0.086, *p* = 0.024), which indicated that single, separated, divorced, or widowed participants were more likely to experience depression than married participants.

### The mediating effect of PA and anxiety

Table 3 tabulated the unstandardized coefficients for the model exploring whether PA and anxiety mediated the relationship between the three perceived neighborhood environmental factors (i.e., accessibility, neighborhood, and pollution) and depression. PA played a mediator role in the relationship between accessibility and depression (*β* = 0.014, *p* = 0.033). PA mediated the relationship between neighborhood and depression (*β* = -0.032, *p* = 0.015). And anxiety mediated neither the association between neighborhood and depression (*β* = -0.085, *p* = 0.100) nor the association between pollution and depression (*β* = -0.117, *p* = 0.120).

**Table 3:** Mediating effects of PA and anxiety in the relationships between perceived neighborhood environment and depression among all participants

Path of mediating effect	Coefficient^a^ (95% *CI*)	*P*
Accessibility → PA → Depression	0.014 (0.001, 0.045)	0.033
Neighborhood → PA → Depression	-0.032 (-0.071, -0.009)	0.015
Neighborhood → Anxiety → Depression	-0.085 (-0.206, 0.025)	0.100
Pollution → Anxiety → Depression	-0.117 (-0.395, 0.048)	0.120

^a^Unstandardized coefficient. *PA* Physical activity, *CI* Confidence interval

## Discussion

In this study, we explored the associations between perceived neighborhood environment and depression, while controlling for socio-demographic characteristics. We found that in mega-communities, residents with depressive symptoms were more likely to report lower satisfaction with neighborhood, lower levels of PA, and higher levels of anxiety. Further, PA mediated the relationships between two perceived neighborhood environmental factors (i.e., accessibility and neighborhood) and depression. Anxiety did not play a mediator role in the relationships between perceived neighborhood environment and depression.

### Depression level

Among the residents of mega-communities in our study, 18.95% of participants reported depressive symptoms, which was higher than the reported depression prevalence among adults nationwide (6.8%) [5] and community residents in Wuhan (6.9%) [62]. Our study did not include residents living in non-mega communities in Guiyang, hampering further comparisons of depression. However, we could make reasonable assumptions based on the characteristics of our participants. Among the individuals who participated in our study, the proportions of unemployed individuals (60.9% vs 48.1% [288.24 million/598.98 million]) and of individuals without local household registration (37.4% vs 27.0% [161.68 million/598.98 million]) were both higher than the proportions of overall residents in Guiyang [63]. Even though we found that employment status and local household registration status were not related to depression among residents in mega-communities in this study, many previous studies have shown that unemployed individuals and individuals without local household registration had a higher risk of depression in the general population [64, 65]. Therefore, we speculated that the prevalence of depression among participants in our study might be higher than that of the general population in Guiyang.

### Perceived neighborhood environment and depression

We found that in mega-communities, residents with depressive symptoms were more likely to be less satisfied with neighborhood that was assessed by the satisfaction with safety, hygiene, parking, greening, lighting, and building shape. Studies of other populations also showed similar results. For example, results of a nationwide cross-sectional survey called “the 2016 China Family Panel Studies” [66] indicated that lower-level depression was associated with better community environment perception which was assessed by community living environment, public facilities, safety, etc. Several cross-sectional studies conducted in Spain and the US have demonstrated that time spent in green space was negatively related to depression level [67, 68]. Another cross-sectional survey conducted in the US reported that residents with more severe community environmental problems such as inadequate garbage collection and limited street lighting were more likely to have higher rates of depression [69]. A possible explanation for the negative association between neighborhood and depression was that, according to the Theory of Planned Behavior, the factors relating to neighborhood might influence health behavior, thus further affecting mental health, including depression [70, 71]. Another possible explanation was that poor neighborhood conditions imposed stress, which could lead to depression [72]. According to the definition of depression, depressive symptoms include persistent low mood and lack of pleasure. Therefore, it was possible that depression might lead to lower satisfaction with neighborhood [1, 2]. Due to the limitation of the design of our study, the causal relationship between perceived neighborhood environment and depression among residents living in mega-communities and the corresponding mechanism needed further research to explore.

### Anxiety and depression

Our study revealed a positive relationship between anxiety and depression, which was consistent with previous studies. Cross-sectional studies conducted among hypertension patients in elderly caring social organizations [32], university students [73, 74], and mothers of young children in fragile families [75] also indicated a positive relationship between anxiety and depression. Previous studies indicated that anxiety might induce depression by causing insomnia and fatigue or impairing individuals’ emotional regulation function and emotional regulation self-efficacy [76, 77].

### PA and depression

Our study showed that PA was inversely associated with depression, which was in line with many previous studies. Several previous cross-sectional studies conducted among Brazilian individuals [78], Japanese older adults [79], and American individuals aged 20 years and older [80] also demonstrated that individuals with lower PA level were more likely to have suffered from depression. Schuch FB et al. conducted a meta-analysis, which included 49 cohort studies and 266,939 individuals, indicating that PA could reduce 17% risk of depression when controlling for potential covariates [81]. On the contrary, low levels of PA might result from depression. A review of several longitudinal studies indicated that depression at baseline might lead to decreased level of PA [77]. The causal relationship between PA and depression needs to be explored by more relevant studies.

### Marital status and depression

Regarding socio-demographic characteristics, we found that married individuals were less likely to suffer from depression, which was consistent with previous findings. In previous studies, marital status was commonly viewed as a key socio-demographic factor that related to depression status. A meta-analysis of cross-sectional studies also indicated that the prevalence of depression in unmarried individuals was higher than that in married individuals [82]. Based on the current results, we could not draw a causal relationship. Marital status might influence the status of depression, and a possible explanation was that unmarried individuals were more likely to experience more loneliness, poorer social support, and lower self-confidence, which were widely considered as risk factors for depression [82]. On the contrary, several studies indicated that depressed couples were more likely to divorce [83, 84]. It seems that depression might affect marital status. Additionally, a longitudinal study in Canada showed that the relationship between marital status and depression was bidirectional [85].

### The mediating effect of PA and anxiety

Our study demonstrated that PA mediated the associations between two factors of perceived neighborhood environment (i.e., accessibility and neighborhood) and depression. Our findings were consistent with previous cross-sectional studies conducted in the US [28] and Hong Kong [30]. A previous study in the US indicated that PA mediated the relationship between neighborhood violence and problems and depressive symptoms [28]. Another study among Hong Kong older residents demonstrated that PA was a mediating variable in the relationship between neighborhood environmental factors and depressive symptoms [30]. The longitudinal National Social Life, Health and Aging Project conducted in the US also found that PA mediated the relationship between urbanicity and depression. The authors explained that exposure to poor neighborhood environment might lead to reduced PA levels, which, in turn, could further lead to increased rates of depression [29]. However, our study could not draw a conclusion on a causal relationship. Another pattern was also possible. For example, depression might lead to reduced PA levels [77], and the depressed residents with lower PA levels were more likely to be dissatisfied with their neighborhood. However, no relevant research has reported this pattern. Future researches may investigate how PA played the mediating role in the relationship between neighborhood environment and depression.

Several studies reported the mediating effect of anxiety in the relationships between non-neighborhood environmental factors (e.g., self-esteem, social support, cyberbullying perpetration) and depression [31–33]. However, the mediating effect of anxiety was not significant in our study. The possible reason was that the mechanism by which anxiety mediated the relationship between non-environmental factors and depression was not suitable for the relationship between environmental factors and depression.

### Strengths and limitations

Our study had several strengths. First, the questionnaire used to assess the perceived neighborhood environment was developed in line with the conditions of communities in mainland China, including 28 factors of neighborhood environment, so that it could comprehensively assess the perceived satisfaction with different neighborhood environmental factors among residents living in mega-communities. Moreover, the reliability and validity of the questionnaire were acceptable [41]. Second, we used SEM that could simultaneously fit the associations among perceived neighborhood environment, PA, anxiety, and depression, with the potential covariates controlled. Meanwhile, SEM also could estimate and test the mediating effects.

Our study also had several limitations. Firstly, as a cross-sectional design, our study could not conclude the causal relationships among perceived neighborhood environment, PA, anxiety, and depression. Secondly, the neighborhood environment was assessed by a self-reported questionnaire, therefore the results might be affected by reporting bias. Thirdly, we did not collect information on non-participants; therefore, we could not know whether there were differences between participants and non-participants. It was not known whether there was a selection bias in our study. Fourthly, with insufficient sample size, we did not estimate the model by community. Fifthly, our findings were based on the residents participating in our study, and might not be suitable to be generalized to other residents of mega-communities. Lastly, several previous studies have indicated that seasonality could affect depression [86–88]. However, our study was carried out between July and August 2022. Therefore, we were unable to control for seasonality as a covariate when we explored the relationship between neighborhood environment and depression.

This was the first study, to the best of our knowledge, to investigate the associations between perceived neighborhood environment and depression in mega-communities in mainland China. The findings of this study indicated that neighborhood, which was assessed by satisfaction with safety, hygiene, parking, greening, lighting, and building shape, was negatively associated with depression. Meanwhile, PA was negatively related to depression, while anxiety was positively related to depression. Additionally, PA mediated the associations between two factors of perceived neighborhood environment (i.e., accessibility and neighborhood) and depression. Anxiety did not mediate the relationships between perceived neighborhood environment and depression.

Cross-sectional studies could not draw causal relationships. Therefore, further research (e.g., multiple-level analysis and prospective cohort study) was needed to explore the relationship between perceived neighborhood environment and depression, especially among unmarried residents living in mega-communities. Meanwhile, important covariates such as seasonal changes should also be included in further research. If further studies confirmed that perceived neighborhood environment could affect depression, community-level interventions promoting satisfaction with characteristics within perceived neighborhood environment (e.g., safety, hygiene, parking, greening, lighting, and building shape) may reduce risk of depression among residents living in mega-communities.

### Supplementary Information

**Additional file 1.**

## Data Availability

The datasets analyzed during the current study are available from the corresponding authors on reasonable request.

## Abbreviations

WHO: World Health Organization
US: United States
PA: Physical activity
PHQ-9: Patients Health Questionnaire-9
GAD-7: Generalized Anxiety Disorder-7
IPAQ-S: Short form of the International Physical Activity Questionnaire
MPA: Moderate-intensity physical activity
VPA: Vigorous-intensity physical activity
MET: Metabolic equivalent
SD: Standard deviation
IQR: Interquartile range
SEM: Structural equation model
CMIN/DF: Discrepancy divided by degree of freedom
RMSEA: Root mean square error of approximation
IFI: Incremental fit index
TLI: Tucker-Lewis incremental fit
CFI: Comparative fit index
NFI: Normed fit index
AGFI: Adjusted goodness of fit index
GFI: Goodness of fit index
BMI: Body mass index
BDNF: Brain-derived neurotrophic factor
