# Supplementary material for: Association between perceived neighborhood environment and depression among residents living in mega-communities in Guiyang, China: a cross-sectional study
Source: BMC Public Health. 2024 Feb 1;24:343. doi: 10.1186/s12889-024-17844-z (PMC10836001; doi:10.1186/s12889-024-17844-z)
Supplement: Supplementary file 1 — Additional file 1. [file 12889_2024_17844_MOESM1_ESM.docx]

**Survey on the perceived neighborhood environment and Mental Health among Residents living in mega-communities in Guiyang City**

**PART 1. Basic Information**

**1.** What is your gender?

① male ② female

**2.** Your date of birth (year-month-day) _________________________

**3.** Is your household registered in Guiyang City?

①Yes ②No

**4.** What is your current occupation?

①Business ②Government staff ③Businessman or self-employed ④Enterprise staff ⑤Worker

⑥Unemployed ⑦Student ⑧Farmer ⑨Other (please specify) _____________________

**5.** What is your highest education level?

①Junior high school or below ②High school or technical school

③College or undergraduate ④Graduate or above

**6.** What is your current marital status?

①Single ②Married ③Widowed ④Divorced ⑤Separated

**7.** What is the per capita monthly income of your family?

①≤2,000 RMB ②2,001-5,000 RMB ③5,001-8,000 RMB

④8,001-15,000 RMB ⑤≥15,001 RMB

**8.** Do you or your family members own this apartment/house?

①Yes ②No

**9.** The number of long-term residents in your family is ______.

**10.** The area of the house you are living in is ________square meters (m2).

**11.** How long have you lived in this community: _______ years.

**PART 2. Satisfaction with neighborhood environment Satisfaction** (Please use "√" to indicate your answer)

| **Items** | **Levels of satisfaction（5 points)** | | | | |
| --- | --- | --- | --- | --- | --- |
|  | **1**  **Strongly dissatisfied** | **2**  **Dissatisfied** | **3**  **Neutral** | **4**  **Satisfied** | **5**  **Strongly satisfied** |
| 1. How satisfied are you with **distance to the main road** in your community |  |  |  |  |  |
| 1. How satisfied are you with **road condition** in your community |  |  |  |  |  |
| 1. How satisfied are you with **sidewalk** in your community |  |  |  |  |  |
| 1. How satisfied are you with **road connectivity** in your community |  |  |  |  |  |
| 1. How satisfied are you with **traffic safety** in your community |  |  |  |  |  |
| 1. How satisfied are you with **traffic congestion** in your community |  |  |  |  |  |
| 1. How satisfied are you with **elevators of residentials building** in your community |  |  |  |  |  |
| 1. How satisfied are you with **doors and windows of residentials building** in your community |  |  |  |  |  |
| 1. How satisfied are you with **building envelop (e.g., external walls, roofs, and floors) of residentials building** in your community |  |  |  |  |  |
| 1. How satisfied are you with **building structure of residentials building** in your community |  |  |  |  |  |
| 1. How satisfied are you with **building facilities (e.g., pipes, pumps, and water tanks) of residentials building** in your community |  |  |  |  |  |
| 1. How satisfied are you with **accessibility to parks and green land** in your community |  |  |  |  |  |
| 1. How satisfied are you with **accessibility to medical institutions** in your community |  |  |  |  |  |
| 1. How satisfied are you with **accessibility to entertainment venues** in your community |  |  |  |  |  |
| 1. How satisfied are you with **accessibility to transportation stations** in your community |  |  |  |  |  |
| 1. How satisfied are you with **accessibility to shopping malls** in your community |  |  |  |  |  |
| 1. How satisfied are you with **neighborhood safety** in your community |  |  |  |  |  |
| 1. How satisfied are you with **neighborhood hygiene** in your community |  |  |  |  |  |
| 1. How satisfied are you with **neighborhood** **parking condition** in your community |  |  |  |  |  |
| 1. How satisfied are you with **neighborhood greening** in your community |  |  |  |  |  |
| 1. How satisfied are you with **neighborbood lighting** in your community |  |  |  |  |  |
| 1. How satisfied are you with **building shape** in your community |  |  |  |  |  |
| 1. How satisfied are you with **indoor noise** in your community |  |  |  |  |  |
| 1. How satisfied are you with **indoor ventilation** in your community |  |  |  |  |  |
| 1. How satisfied are you with **indoor temperature** in your community |  |  |  |  |  |
| 1. How satisfied are you with **indoor light** in your community |  |  |  |  |  |
| 1. How satisfied are you with **air pollution** in your community |  |  |  |  |  |
| 1. How satisfied are you with **other pollution (e.g., water)** in your community |  |  |  |  |  |

**PART 3. Patient health questionnaire(PHQ-9)** (Over the last 2 weeks, how often have you been bothered by any of the following problems? use "√" to indicate your answer)

|  | **0**  **Not at all** | **1**  **Several days** | **2**  **More than half the days** | **3**  **Nearly**  **everyday** |
| --- | --- | --- | --- | --- |
| 1. Little interest or pleasure in doing things |  |  |  |  |
| 2. Feeling down, depressed, or hopeless |  |  |  |  |
| 3. Trouble falling or staying asleep, or sleeping too much |  |  |  |  |
| 4. Feeling tired or having little energy |  |  |  |  |
| 5. Poor appetite or overeating |  |  |  |  |
| 6. Feeling bad about yourself or that you are a failure or have let yourself or your family down |  |  |  |  |
| 7. Trouble concentrating on things, such as reading the newspaper or watching television |  |  |  |  |
| 8. Moving or speaking so slowly that other people could have noticed. Or the opposite being so figety or restless that you have been moving around a lot more than usual |  |  |  |  |
| 9. Thoughts that you would be better off dead, or of hurting yourself |  |  |  |  |

**PART 4. General Anxiety Disorder-7 (GAD-7)** (Over the last 2 weeks, how often have you been bothered by any of the following problems? use "√" to indicate your answer)

|  | **0**  **Not at all** | **1**  **Several days** | **2**  **More than half the days** | **3**  **Nearly**  **everyday** |
| --- | --- | --- | --- | --- |
| 1. Feeling nervous, anxious, or on edge |  |  |  |  |
| 2. Not being able to stop or control worrying |  |  |  |  |
| 3. Worrying too much about different things |  |  |  |  |
| 4. Trouble relaxing |  |  |  |  |
| 5. Being so restless that it is hard to sit still |  |  |  |  |
| 6. Becoming easily annoyed or irritable |  |  |  |  |
| 7. Feeling afraid, as if something awful might happen |  |  |  |  |

**PART 5. International physical activity questionnaire shor-form (IPAQ-S)** (We are interested in finding out about the kinds of physical activities that people do as part of their everyday lives. The questions will ask you about the time you spent being physically active in the last 7 days. Please answer each question even if you do not consider yourself to be an active person. Please think about the activities you do at work, as part of your house and yard work, to get from place to place, and in your spare time for recreation, exercise or sport.)

Think about all the **vigorous** activities that you did in the last 7 days. Vigorous physical activities refer to activities that take hard physical effort and make you breathe much harder than normal. Think only about those physical activities that you did for at least 10 minutes at a time.

1. During the **last 7 days**, on how many days did you do vigorous physical activities like heavy lifting, digging, aerobics, or fast bicycling?

**_____ days per week**

□No vigorous physical activities  **→**  Skip to question 3

1. How much time did you usually spend doing **vigorous** physical activities on one of those days?

**_____ hours per day**

**_____ minutes per day**

□Don’t know/Not sure

Think about all the moderate activities that you did in the last 7 days. Moderate activities refer to activities that take moderate physical effort and make you breathe somewhat harder than normal. Think only about those physical activities that you did for at least 10 minutes at a time.

1. During the **last 7 days**, on how many days did you do moderate physical activities like carrying light loads, bicycling at a regular pace, or doubles tennis? Do not include walking.

**_____ days per week**

□No moderate physical activities → Skip to question 5

4. How much time did you usually spend doing moderate physical activities on one of those

days?

**_____ hours per day**

**_____ minutes per day**

□Don’t know/Not sure

Think about the time you spent **walking** in the **last 7 days**. This includes at work and at home, walking to travel from place to place, and any other walking that you have done solely for recreation, sport, exercise, or leisure.

5. During the **last 7 days**, on how many days did you **walk** for at least 10 minutes at a time?  **_____ days per week**

□No walking → Skip to question 7

6. How much time did you usually spend **walking** on one of those days?

**_____ hours per day**

**_____ minutes per day**

□Don’t know/Not sure

The last question is about the time you spent **sitting** on weekdays during the **last 7 days**. Include time spent at work, at home, while doing course work and during leisure time. This may include time spent sitting at a desk, visiting friends, reading, or sitting or lying down to watch television.

7. During the **last 7 days**, how much time did you spend **sitting** on a **week day**?

**_____ hours per day**

**_____ minutes per day**

□Don’t know/Not sure

**This is the end of the questionnaire. Thank you for your generous help!**

The following information will be filled by the interviewers.

Date:

Place:

Questionnaire No.____________

Interviewers: ________________
